# Supplementary figures and images for: Genes Containing Long Introns Occupy Series of Bands and Interbands in Drosophila melanogaster Polytene Chromosomes
Source: Genes (Basel). 2020 Apr 11;11(4):417. doi: 10.3390/genes11040417 (PMC7230524; doi:10.3390/genes11040417)

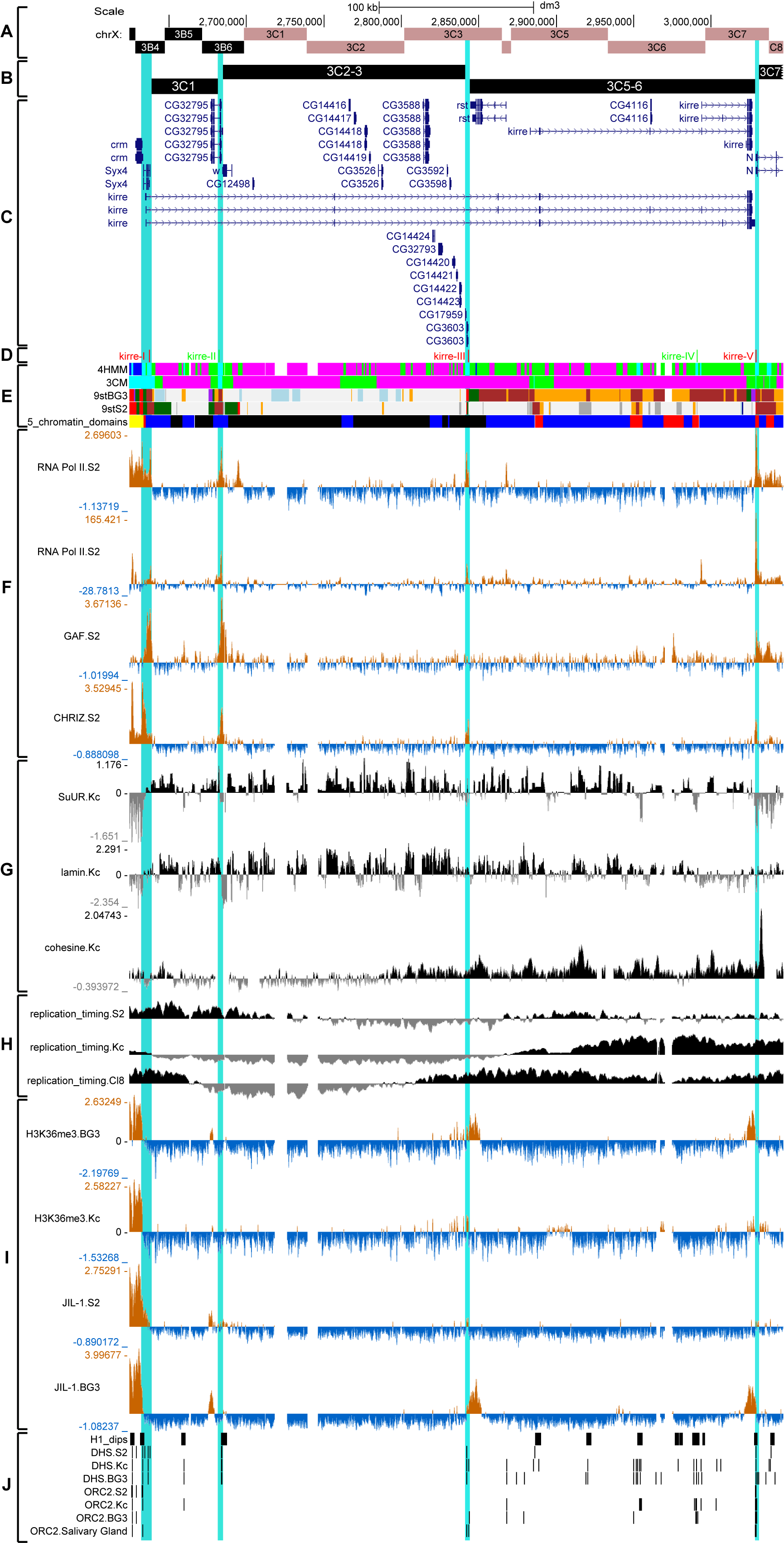

Supplement: Supplementary file 1 [file genes-11-00417-s001.zip › Supplementary Figures and Tables/Fig. S1 kirre full passport.tif]
